# Septoplasty with or without postoperative nasal packing? Prospective study

**DOI:** 10.5935/1808-8694.20130084

**Published:** 2015-10-08

**Authors:** Maria Teresa Bernardo, Sandra Alves, Nuno Barros Lima, Diamantino Helena, Artur Condé

**Affiliations:** a MD. (Intern at the Otolaryngology Ward - Vila Nova de Gaia Hospital Centre/Espinho. EPE).; b ENT Physician (Hospital Assistant - Vila Nova de Gaia Hospital Centre/Espinho. EPE).; c ENT Physician (Graduate Hospital Assistant - Vila Nova de Gaia Hospital Centre/Espinho. EPE).; d ENT Physician (Department Head - Vila Nova de Gaia Hospital Centre/Espinho. EPE).; Hospital Centre of Vila Nova de Gaia/Espinho. EPE.

**Keywords:** internal nasal packing, nasal septum, quality of life

## Abstract

Anterior nasal packing is carried out in a number of nasal surgeries, especially in septoplasty. However, it is not an innocuous procedure and for this its benefit has been challenged.

**Objective:**

To assess the need for anterior nasal packing and the quality of life of patients submitted to septoplasty.

**Method:**

Patients submitted to septoplasty with or without inferior turbinoplasty were randomized to receive or not anterior nasal packing postoperatively. We recorded and compared postoperative data (pain and bleeding). Quality of life was assessed before and after surgery. This is a randomized prospective study.

**Results:**

We had 73 patients (37 packed and 36 who did not receive a nasal packing) with a minimum follow-up of 3 months. Patients with nasal packing complained more of nasal pain and headache in the immediate postoperative period. Of these patients, 75.7% reported moderate/intense pain upon nasal packing removal. Bleeding was more frequent in those patients who did not receive a nasal packing, and only 1 patient required packing. All the patients enjoyed an improvement in quality of life.

**Conclusion:**

Septoplasty improves the quality of life of patients with septal deviation and nasal obstruction. Routine use of anterior nasal packing should be challenged for not presenting proven benefit.

## INTRODUCTION

Anterior nasal packing is done routinely in many nasal surgeries, particularly in septoplasty. It aims at preventing postoperative onset of bleeding, septal hematoma or nasal synechia, ensuring mucoperichondrium flap coaptation and cartilage stabilization in order to get the best surgical results^1^, ^2^, ^3^, ^4^. However, there is no scientific evidence to support its benefit, and anterior nasal packing is not an innocuous procedure^1^. Its brings about discomfort/pain (especially upon removal), nasal mucosa trauma, epiphora, local infection, discomfort in swallowing, sleep disturbances and, very rarely, toxic shock, displacement with aspiration and vagal reflex^5^. Its benefit has been challenged and its routine use started to be questioned and often not recommended^1^, ^6^, ^7^, ^8^. Several recent studies have been carried out to assess the relevance of anterior nasal packing with different surgical and packing techniques, which makes it difficult to compare its use in our day-to-day.

This study aims at assessing the need for anterior nasal packing in patients submitted to septoplasty in our department, comparing the incidence of postoperative complications in patients who received nasal packing and those who did not. We also evaluated the surgical results in terms of quality of life comparing the two groups before and after surgery.

## METHOD

We did a prospective and randomized study, approved by the Hospital Ethics Committee (No. 97/2010), with patients undergoing septoplasty with or without inferior turbinoplasty between September of 2009 and February of 2010. Patients were packed at previously defined alternate months, regardless of the type of septal deviation or bleeding during surgery. Thus, we ended up having two groups of homogeneous patients with and without anterior nasal packing. We excluded patients younger than 15 years; those submitted to revision surgeries; those with other associated nasal surgeries; those with uncontrolled hypertension; coagulation disorders (including iatrogenic) and those in whom it was strictly necessary to pack during surgery.

Septoplasty was performed according to the modified Cottle technique after injection with 2% lidocaine and epinephrine 1:200000. The inferior turbinate mucosa underwent electrocauterization when deemed necessary. In all the patients we placed Shah (Exmoor^®^) nasal plates with transfixing suture (3.0 silk wire) to cover all of the cartilaginous portion of the septum. In packed patients we used a stringed-nasal packing (Ivalon^®^) impregnated with antibiotic ointment (Terricil^®^). Postoperatively, we prescribed antibiotics (amoxicillin and clavulanic acid - 875 mg 125 mg bid) for 8 days, analgesia when needed and saline nasal flushing. The nasal packings were removed (after injecting saline solution in the packing) and discharged after 48 hours. The nasal plates were removed in an outpatient basis between the 7^th^ and 10^th^ postoperative day.

We collected data on the surgical procedure (inferior turbinoplasty, amount of bleeding by measuring the volume of the vacuum bag minus the saline solution volume used in flushing and surgery duration); postoperative pain (headache and nasal) based on the visual analogue scale (VAS 0-10) at 24 and 48 hours, and all complications, including hemorrhage, epiphora, discomfort upon swallowing, sleep disorder, septal hematoma, infection and adhesions. We assessed their quality of life before surgery and three months afterwards using the NOSE questionnaire (questionnaire to assess quality of life of patients with nasal pathology from the American Academy of Otolaryngology, validated in Portuguese for our population^9^) upon admission and by telephone.

The data was statistically analyzed using the SPSS version 17.0. To evaluate group homogeneity we used the *Student's t*-test to compare the mean ages, and the Chi-square test to compare the incidence of male patients. To compare the two groups with regards to nasal pain, headache and the NOSE score at 3 months we used the Mann-Whitney test. We used the chi-square test to compare the percentage of hemorrhage, epiphora, discomfort upon swallowing and sleep disturbances in the two groups. To compare patients subjected or not to inferior turbinoplasty with regards to hemorrhage and postoperative pain we used the Ficher and the Mann-Whitney tests, respectively. Finally, the comparison of the different parameters of the NOSE questionnaire before and after surgery was carried out using the Wilcoxon test. We considered a *p* of 0.05 (α = 0.05) as statistically significant.

## RESULTS

We had 95 patients with these conditions and took 22 off because it was not possible to complete the NOSE survey postoperatively with them. The remaining 73 patients, 64.4% males and with a mean age of 40.1 ± 13.6 years (minimum 16, maximum 67) were divided into two homogeneous groups, 37 received nasal packing and 36 did not. The minimum follow-up was 3 months. The mean duration of surgery was 36.6 minutes (minimum 15, maximum 60) and inferior turbinoplasty was performed in 70% of the patients. The average intraoperative bleeding was 22.6 ml (5 ml minimum, maximum 100 ml).

Nasal pain and headache at 24 and 48 hours were higher in patients with nasal packing although not statistically significant, except in nasal pain at 48 hours (Table 1). The average pain by the VAS upon nasal packing removal was 5.65, with 75.7% of the patients reporting a value greater than 3 - moderate to severe pain.

**Table 1:** Comparison of the VAS median of postoperative pain in patients with and without postoperative anterior nasal packing.

	With packing n = 37	Without packing n = 36	*p* value*
Nasal pain at 24h	4	2.5	183
Nasal pain at 48h	4	1	43
Headache at 24h	1	0	168
Headache at 48h	2	0	0.05

Mann-Whitney test.

With respect to post-operative bleeding, it was more frequent in patients without nasal packing (*p* > 0.05) (Table 2) with a relative risk of 2.81 and a risk attributable to the lack of nasal packing of 0.19. It was not possible to quantify the bleeding, but only one patient needed packing at the 6^th^ postoperative day (*p* > 0.05).

**Table 2:** Comparison of postoperative nasal hemorrhage in patients with and without nasal packing.

	With nasal packing* (%)	Without nasal packing** (%)	*p* value***
Postoperative hemorrhage	4 (10.8)	11 (30.6)	78

* n = 37;

** n = 36;

*** Chi-squared test.

The patients submitted to inferior turbinate cauterization had higher incidence of postoperative bleeding (*p* < 0.05), but did not have more pain (*p* > 0.05).

Epiphora, discomfort in swallowing and sleep disturbances were more frequent in patients with nasal packing, but only epiphora reached statistical significance (Table 3). There were no patients with septal hematoma, adhesions or local infection.

**Table 3:** Comparison of the incidence of epiphora, swallowing discomfort and sleep disorders in patients with and without nasal packing.

	With nasal packing* (%)	Without nasal packing** (%)	*p* value***
Epiphora	15 (40.5)	1 (2.8)	0
Swallowing discomfort	8 (21.6)	4 (11.1)	226
Sleep disorder	23 (62.2)	17 (47.2)	0.2

* n = 37;

** n = 36;

*** Chi-square test.

In comparing the preoperative NOSE values and 3 months after surgery, there were improvements in all parameters (*p* < 0.05). There was no difference in NOSE scores at 3 months between the two groups of patients, with and without nasal packing (*p* > 0.05).

## DISCUSSION

Nasal pain and headache were higher in patients with nasal packing, but this was not statistically significant except for nasal pain at 48 hours. The lack of statistical significance may be explained by not having taken into account the septal deviation size (the higher, the greater the need for bone manipulation, thus more pain) and it was not possible to standardize the size of the nasal packing used since each surgeon uses as much as deemed necessary. In any event, pain upon nasal packing removal (present only in patients with nasal packing) is also an important factor to consider. As far as pain is concerned, although not statistically significant, it is clinically important. This was also demonstrated in the study by Cukurov et al.^10^, in which postoperative pain in 697 patients submitted to septoplasty was also evaluated based on VAS (Visual Analogue Scale). This study showed that patients submitted to nasal packing had more pain than those in which only a suture was used to ensure coaptation of the mucoperichondrium flaps.

Postoperative bleeding was more frequent in patients without nasal packing: however this data was obtained subjectively based on patient information through the telephone survey. The risk attributable to the fact of not being packed was 0.19, and only one patient required nasal packing. Therefore, patients usually have less hemorrhage without postoperative anterior nasal packing. The association of lower turbinoplasty increases the risk of bleeding. Probably if these patients had been excluded we would have had fewer bleeding episodes. Theoretically, if one manages to get a good bleeding control during surgery, postoperative bleeding is not significant^4^, ^11^. If, at the end of surgery, nasal packing is needed, the use of a resorbable material will always be a good option.

The remaining complications were more frequent in patients with nasal packing and contributed to greater morbidity in the immediate postoperative period in these patients. Epiphora results from lacrimal duct obstruction; discomfort in swallowing manifests mainly at the ear level problem and sleep disorders happen because of worsening in apnea and consequent frequent awakenings.

There were no septal hematomas, adhesions or local infections, probably due to the systematic use of nasal plates and postoperative antibiotics.

Septoplasty improved nasal breathing and quality of life for all patients when assessed 3 months after surgery, with no differences between the two groups. We can therefore infer that nasal packing does not guarantee better surgical outcomes. Quality of life improvements measured through the NOSE questionnaire of patients submitted to septoplasty had already been proven by Bezerra et al.^12^.

There are now many published studies that question the need for anterior nasal packing^1^, ^4^, ^8^, ^13^, ^14^, but the wide variety of materials and techniques used make it difficult to extrapolate them to a day-to-day basis. This study, developed in our department based on our clinical practice, held a change of action for a non-systematic use of anterior nasal packing.

## CONCLUSION

Septoplasty with or without inferior turbinoplasty improves the quality of life of patients with nasal septum deviation independent of using postoperative anterior nasal packing. Routine anterior nasal packing should be challenged for not presenting proven benefit, increasing morbidity and it can potentially cause serious complications.
